# Predictors of success in establishing orthotopic patient-derived xenograft models of triple negative breast cancer

**DOI:** 10.1038/s41523-022-00502-1

**Published:** 2023-01-10

**Authors:** Gloria V. Echeverria, Shirong Cai, Yizheng Tu, Jiansu Shao, Emily Powell, Abena B. Redwood, Yan Jiang, Aaron McCoy, Amanda L. Rinkenbaugh, Rosanna Lau, Alexander J. Trevarton, Chunxiao Fu, Rebekah Gould, Elizabeth E. Ravenberg, Lei Huo, Rosalind Candelaria, Lumarie Santiago, Beatriz E. Adrada, Deanna L. Lane, Gaiane M. Rauch, Wei T. Yang, Jason B. White, Jeffrey T. Chang, Stacy L. Moulder, W. Fraser Symmans, Susan G. Hilsenbeck, Helen Piwnica-Worms

**Affiliations:** 1 Department of Experimental Radiation Oncology, University of Texas MD Anderson Cancer Center, Houston, TX 77030 USA; 2 Department of Pathology, University of Texas MD Anderson Cancer Center, Houston, TX 77030 USA; 3 Department of Breast Medical Oncology, University of Texas MD Anderson Cancer Center, Houston, TX 77030 USA; 4 Department of Breast Imaging, University of Texas MD Anderson Cancer Center, Houston, TX 77030 USA; 5 Department of Abdominal Imaging, University of Texas MD Anderson Cancer Center, Houston, TX 77030 USA; 6 Department of Integrative Biology and Pharmacology, University of Texas Health Science Center, Houston, TX 77030 USA; 7 Lester and Sue Smith Breast Center, Baylor College of Medicine, Houston, TX 77030 USA; 8 Present Address: Lester and Sue Smith Breast Cancer Center and Department of Medicine, Baylor College of Medicine, Houston, TX 77030 USA

**Keywords:** Breast cancer, Cancer models

## Abstract

Patient-derived xenograft (PDX) models of breast cancer are an effective discovery platform and tool for preclinical pharmacologic testing and biomarker identification. We established orthotopic PDX models of triple negative breast cancer (TNBC) from the primary breast tumors of patients prior to and following neoadjuvant chemotherapy (NACT) while they were enrolled in the ARTEMIS trial (NCT02276443). Serial biopsies were obtained from patients prior to treatment (pre-NACT), from poorly responsive disease after four cycles of Adriamycin and cyclophosphamide (AC, mid-NACT), and in cases of AC-resistance, after a 3-month course of different experimental therapies and/or additional chemotherapy (post-NACT). Our study cohort includes a total of 269 fine needle aspirates (FNAs) from 217 women, generating a total of 62 PDX models (overall success-rate = 23%). Success of PDX engraftment was generally higher from those cancers that proved to be treatment-resistant, whether poorly responsive to AC as determined by ultrasound measurements mid-NACT (*p* = 0.063), RCB II/III status after NACT (*p* = 0.046), or metastatic relapse within 2 years of surgery (*p* = 0.008). TNBC molecular subtype determined from gene expression microarrays of pre-NACT tumors revealed no significant association with PDX engraftment rate (*p* = 0.877). Finally, we developed a statistical model predictive of PDX engraftment using percent Ki67 positive cells in the patient’s diagnostic biopsy, positive lymph node status at diagnosis, and low volumetric reduction of the patient’s tumor following AC treatment. This novel bank of 62 PDX models of TNBC provides a valuable resource for biomarker discovery and preclinical therapeutic trials aimed at improving neoadjuvant response rates for patients with TNBC.

## Introduction

Chemotherapy and the recently approved PD1 inhibitor pembrolizumab are the recommended standard of care for patients with primary triple negative breast cancer (TNBC). While nearly 50% of TNBC patients treated with standard neoadjuvant (pre-surgical) chemotherapy (NACT) have excellent response, the extent of residual cancer burden (RCB) in the remainder of patients is strongly associated with heightened risk of distant metastasis and death^1^. Thus, overcoming insensitivity to chemotherapy will improve survival for patients with TNBC. TNBC encompasses a heterogeneous population of tumors exhibiting diverse histologic^2^, genomic^3^, and transcriptomic^4^ profiles. This inter-patient heterogeneity necessitates generation of appropriate experimental models capturing the diverse features of TNBC.

Patient-derived xenograft (PDX) models of breast cancer have been an invaluable tool with which to conduct preclinical pharmacologic testing^5–9^, delineate mechanisms of therapy resistance^10,11^, investigate metastasis^12–17^, and catalog genomic complexity^18–20^. PDX models of TNBC have been established by several groups using a variety of methods (reviewed in ref. ^21^), usually involving engraftment of frozen or fresh core needle biopsy-derived tumor fragments or dissociated tumor cells into mammary glands of immune-compromised mice. Rates of successful PDX engraftment vary among these studies, and it is unclear which, if any, clinical or molecular features are consistently associated with success in PDX establishment. Kaplan-Meier analysis of overall survival revealed a significant correlation with successful PDX engraftment in a cohort of 24 newly diagnosed breast cancers, some of which were TNBC^22^. To better understand features that predict successful engraftment, we leveraged a large biobank of orthotopic TNBC PDX models from patient tumors that exhibited differential responses to NACT and that encompass different molecular subtypes of TNBC, determined at the time of diagnosis.

We generated PDX models from the tumors of TNBC patients enrolled on a neoadjuvant clinical trial, ARTEMIS (A Robust TNBC Evaluation fraMework to Improve Survival (NCT02276443)). Biopsies were obtained by fine-needle aspiration (FNA) prior to treatment with chemotherapy (‘pre’), following four cycles of Adriamycin combined with cyclophosphamide (AC; ‘mid’), and following treatment with a taxane, sometimes combined with an experimental targeted therapy prior to surgery (‘post’). We catalogued clinical information associated with each patient biopsy and conducted statistical analyses to search for parameters associated with successful PDX engraftment and we demonstrated that FNAs can be used to generate PDX models. Due to its minimally invasive nature, FNA enabled serial sampling of patient tumors throughout the course of NACT. Our PDX cohort includes models derived from tumors encompassing a broad heterogeneity of chemotherapy responses, histologic features, and molecular TNBC subtypes.

## Results

### Establishment and validation of PDX models

**Fig. 1:**
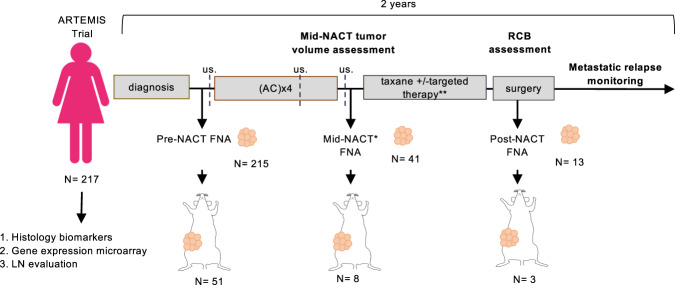
Establishment of PDX models in alignment with a neoadjuvant clinical trial for TNBC. 217 patients with TNBC were biopsied for PDX generation. Pre-NACT biopsies were stored for FFPE and nucleic acid evaluation. Biopsies for were obtained pre-, mid-, and post-NACT. Tumor volume was measured by ultrasound (us). All biopsies used for PDX engraftment were FNAs. LN, lymph node. Patients with obvious residual tumor mass following four cycles of AC were biopsied for mid-NACT PDX. **Targeted therapies were administered based on pre-NACT biomarker status.

A total of 269 tumor samples were obtained from 217 patients enrolled in the ARTEMIS clinical trial between November 2015 and December 2017 (Fig. 1). Tumor cells from three aspirates were pooled and engrafted into the fourth mammary fat pads (MFPs) of non-obese diabetic/severe combined immunodeficient (NOD/SCID) mice. Our previous histologic, genomic, and transcriptomic comparisons of FNA-derived PDX tumors with the originating patient tumor revealed a high degree of concordance^10,12^, demonstrating that FNAs are a suitable biopsy method for PDX establishment. Tumor cells were immediately transferred from the clinic to the lab for digestion into single cells, cell counting, and orthotopic engraftment. Following laboratory processing, the number of viable cells obtained from pooled FNAs ranged from 725 cells to 1.8 million cells (Supplementary Table 1). There was a non-significant trend toward correlation between the number of viable cells implanted and success in PDX establishment (*p* = 0.097). With this sample size, we had 80% power (two-tailed alpha = 5%) to detect standardized effects sizes of 0.45.

PDX models were considered established after two consecutive passages in mice (P2) and if they passed three validation tests. Of 269 engrafted FNAs, 62 resulted in successful PDX establishment, for an overall take-rate of 23%. Each model was validated by: (1) short-tandem repeat DNA fingerprinting to confirm the unique profile and purity of each tumor model, (2) quantitative PCR (qPCR) with human and mouse-specific genomic DNA probes to confirm the human origin of tumor cells, and (3) PCR to confirm absence of GFP, present in immortalized human stromal fibroblasts that were used to pre-humanize the fourth mammary pads of mice prior to engraftment of patient tumor cells at passage zero^23^. qPCR demonstrated that the ratio of human to mouse genomic DNA was heterogeneous between PDX models but was usually consistent across passages within a given model (Supplementary Table 1).

### Analysis of clinical features associated with PDX engraftment

**Fig. 2:**
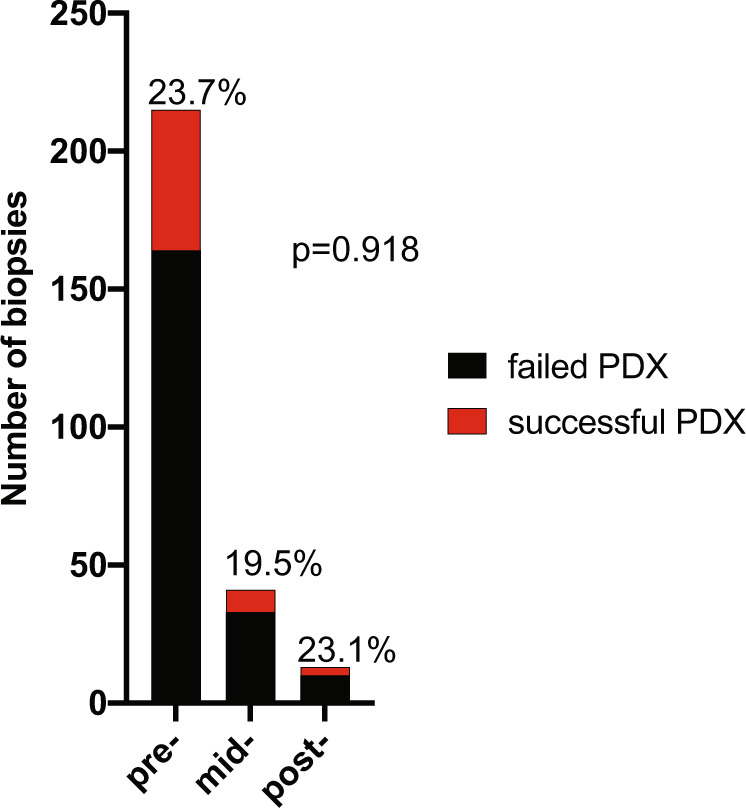
PDX establishment from pre-, mid-, and post-NACT biopsies. The number of patient biopsies obtained is displayed and the percentage of successful PDXs generated from each treatment stage is written atop each bar. The *p* value was computed by Fisher’s exact test.

**Fig. 3:**
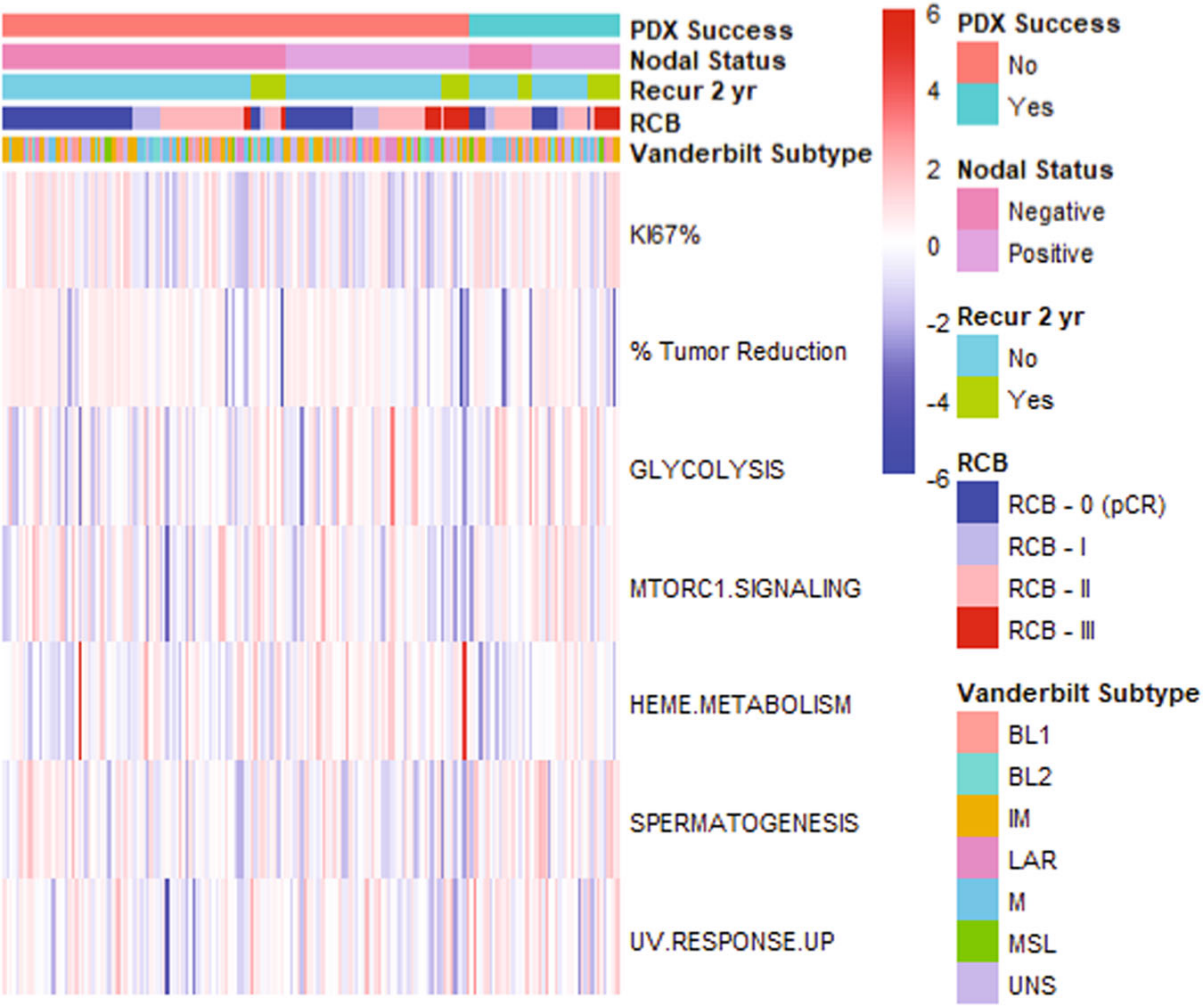
Continuous variables associated with PDX success. Continuous variables associated with PDX success are included in this heatmap.

Of the 62 established PDX models (*n* = 269 biopsies were received in total), 51 were from pre-NACT biopsies (of *n* = 215 total received), 8 were from mid-therapy biopsies (of *n* = 41 total received), and 3 were from post-therapy biopsies (of *n* = 13 total received) (Fig. 2). These results demonstrate that FNAs from tumors prior to, during, or upon completion of NACT can be used to successfully generate PDX models. Treating each biopsy as an independent observation, there was no association between treatment biopsy time point and tumor take-rate (*p* = 0.918, Table 1; Fig. 2). However, considering paired biopsies, we did observe that successful pre-NACT biopsy PDX establishment was positively associated with mid- or post-treatment biopsy PDX success from the same patient (Fisher’s exact test, *p* = 0.0012). We next conducted univariate analyses of clinical and gene expression features, focusing all downstream analyses on only the pre-NACT biopsies to avoid possible confounding effects of repeated sampling of the same patient. The number of patients with available data for each variable are indicated in Supplementary Fig. 1. Univariate analyses of other baseline clinical features, including stage at diagnosis, tumor volume at diagnosis, race, and ethnicity, revealed no correlations with success of PDX establishment (Table 1). However, both positive lymph node status at diagnosis, as well as higher percent Ki67 positive cells by immuno-histochemical (IHC) analysis of diagnostic FFPE specimens, were significantly associated with successful PDX establishment (*p* = 0.020 and *p* = 0.032, respectively; Table 1, Fig. 3). Other histologic analyses of diagnostic FFPE samples, including histologic classification and IHC for vimentin or Androgen Receptor (AR), revealed no associations with success of PDX establishment (Table 1).

**Table 1:** Clinical variables of pre-NACT biopsies analyzed for correlation with PDX success.

	Failed PDXs *n* (%)	Successful PDXs *n* (%)	Success rate (%)	*p* value
*Categorical variables*				
Biopsy stage (*n* = 269)				
Pre	164 (79.2)	51 (82.3)	23.7	0.918
Mid	33 (15.9)	8 (12.9)	19.5	
Post	10 (4.8)	3 (4.8)	23.1	
Race (*n* = 215)				
Asian	12 (7.3)	3 (5.9)	20.0	0.875
Black	24 (14.6)	10 (19.6)	29.4	
White	107 (65.2)	32 (62.7)	23.0	
Unknown	21 (12.8)	6 (11.8)	22.2	
Ethnicity (*n* = 215)				
Hispanic	29 (17.7)	9 (17.6)	23.7	1
Non-Hispanic	124 (75.6)	39 (76.5)	23.9	
Unknown	11 (6.7)	3 (5.9)	21.4	
Stage at diagnosis (*n* = 203)				
I	18 (11.7)	3 (6.1)	14.3	0.127
II	101 (65.6)	28 (57.1)	21.7	
III	35 (22.7)	18 (36.7)	34.0	
Lymph node status at diagnosis (*n* = 203)				
Negative	94 (61.0)	20 (40.8)	17.5	0.02
Positive	60 (39.0)	29 (59.2)	32.6	
Histology class (*n* = 203)				
Invasive ductal carcinoma	131 (85.1)	41 (83.7)	23.8	0.942
Metaplastic	15 (9.7)	5 (10.2)	25.0	
Other	8 (5.2)	3 (6.1)	27.3	
RCB status at surgery (*n* = 200)				
pCR	68 (44.4)	14 (29.8)	17.1	0.215
RCB I	20 (13.1)	5 (10.6)	20.0	
RCB II	48 (31.4)	20 (42.6)	36.8	
RCB III	17 (11.1)	8 (17.0)	32.0	
Grouped RCB status (*n* = 200)				
pCR + RCB I	88 (57.5)	19 (4.4)	17.8	0.046
RCB II + RCB III	65 (42.4)	28 (59.6)	30.1	
Two-year relapse (*n* = 203)				
No	134 (87.0)	34 (69.4)	20.2	0.008
Yes	20 (13.0)	15 (30.6)	42.9	
TNBC subtype (*n* = 207)				
BL1	28 (17.9)	13 (25.5)	31.7	0.877
BL2	16 (10.3)	5 (9.8)	23.8	
IM	36 (23.1)	10 (19.6)	21.7	
LAR	16 (10.3)	3 (5.9)	15.8	
M	26 (16.7)	10 (19.6)	27.8	
MSL	10 (6.4)	2 (3.9)	16.7	
UNS	24 (15.4)	8 (15.7)	25.0	
Vimentin % positivity (IHC, *n* = 197)				
0–<1%	56 (37.3)	15 (31.9)	21.1	0.745
1–10%	46 (30.7)	13 (27.7)	22.0	
11–25%	13 (8.7)	7 (14.9)	35.0	
26–49%	10 (6.7)	5 (10.6)	33.3	
50–75	17 (11.3)	5 (10.6)	22.7	
>75%	8 (5.3)	2 (4.3)	20.0	
AR % positivity (IHC, *n* = 197)				
0%	41 (27.3)	22 (46.8)	34.9	0.257
<1%	37 (24.7)	10 (21.3)	21.3	
1–9%	20 (13.3)	6 (12.8)	23.1	
10–25%	24 (16.0)	5 (10.6)	17.2	
26–50%	11 (7.3)	3 (6.4)	21.4	
51–75%	3 (2.0)	0 (0.0)	0.0	
>75%	14 (9.4)	1 (2.1)	6.7	

**Continuous variables**	**Mean (SD)**	**Mean (SD)**		
% tumor reduction after AC (*n* = 200)	61.89 (55.35)	35.18 (92.59)		0.063
Ki67 % positivity (IHC; *n* = 197)	58.02 (27.26)	66.53 (22.12)		0.037
ssGSEA score: glycolysis (*n* = 207)	0.256 (0.028)	0.265 (0.025)		0.027
ssGSEA score: MTORC1 signaling (*n* = 207)	0.450 (0.029)	0.458 (0.024)		0.046
ssGSEA score: heme metabolism (*n* = 207)	0.225 (0.018)	0.220 (0.013)		0.051
ssGSEA score: spermatogenesis (*n* = 207)	−0.134 (0.041)	−0.121 (0.039)		0.030
ssGSEA score: UV response up (*n* = 207)	0.261 (0.020)	0.269 (0.020)		0.013

### Association of patient response to NACT and pre-NACT PDX engraftment

Next, we asked if clinically chemoresistant tumors were more likely to generate PDX models than were chemosensitive tumors. The ARTEMIS trial collected ultrasound measurements to estimate tumor volume reduction following completion of front-line AC (‘mid-therapy’). The correlation of tumor volume reduction post-AC, as measured by ultrasound, with PDX success (considering only pre-NACT PDXs) trended toward statistical significance (*p* = 0.063; Table 1). Following completion of NACT, tumors were surgically resected, and pathological evaluation of the breast and lymph nodes was conducted to assess RCB status. A Fisher’s Exact Test comparing pre-NACT biopsy PDX success by RCB categories found no association (*p* = 0.215; Table 1). However, dichotomizing RCB into pCR or RCB I compared to RCB II or III (Table 1) revealed a modest association with higher RCB (*p* = 0.046, Fisher’s exact test; Table 1). In this cohort, 203 patients had 2-year follow-up information, and 17% of those patients had metastatic relapse within 2 years of surgery (*n* = 35). Tumors from patients with metastatic relapse within 2 years of surgery were more than twofold more likely to generate a PDX model (considering only pre-NACT biopsies) than were tumors from patients who were still relapse-free 2 years after resection (*p* = 0.008, Fisher’s exact test; Table 1). Taken together, these results indicate that in TNBC, early metastatic relapse is a stronger predictor of PDX success than response to NACT as measured by mid-therapy ultrasound determined reduction in tumor size or by RCB evaluation at the time of surgery.

### Association of pre-NACT biopsy gene expression profiles with PDX engraftment

Of the 217 ARTEMIS patients biopsied for PDX establishment, 207 pre-NACT biopsies passed quality control for Affymetrix gene expression microarray analysis. To determine the likelihood of each TNBC transcriptomic subtype to successfully give rise to a PDX, we used TNBCtype^4,24^ to predict the subtypes of the 207 diagnostic patient biopsies. The subtype distribution of biopsies in this cohort was similar to that observed in previous studies^4^. No association was detected between TNBC subtype assignment and PDX success rate (*p* = 0.877; Fig. 3).

To determine if gene expression profiles were associated with PDX success, we computed scores for several clinically relevant gene expression signatures from patients’ microarray gene expression profiles. This was an exploratory analysis and thus *p* values were not corrected for multiple comparisons. Scoring for an epithelial-mesenchymal transition (EMT) signature^25^, embryonic plasticity signature^26^, and PIK3CA pathway activation revealed no significant association with PDX establishment (Supplementary Table 2). We next computed scores from a curated set of 50 Hallmarks^27^ pathways for each individual biopsy using single sample gene set enrichment analysis (ssGSEA) and found that several pathways, including glycolysis (*p* = 0.027) and MTORC1 signaling (*p* = 0.046), were moderately univariately associated with successful PDX engraftment (Supplementary Table 2; Fig. 3). Hallmarks signatures had within-group standard deviations of ~0.04 (range 0.01–0.10) on average, so differences in mean scores of about ±0.018 were detectable with high reliability. Despite reaching statistical significance, the difference in magnitude of ssGSEA pathway scores was small, indicating that biological validation of the role of these pathways in the success of PDX engraftment will require further experimentation. In addition, pre-NACT biopsy PDX success did not associate with tumor-infiltrating lymphocyte (TIL) gene expression signature assessed in the gene expression data of pre-NACT biopsies from patients (*p* = 0.980; Supplementary Table 2). Similarly, stromal TILs in pre-NACT tumor FFPE samples from these patients were not associated with PDX success (*p* = 0.431).

### A statistical model predicting PDX success

**Fig. 4:**
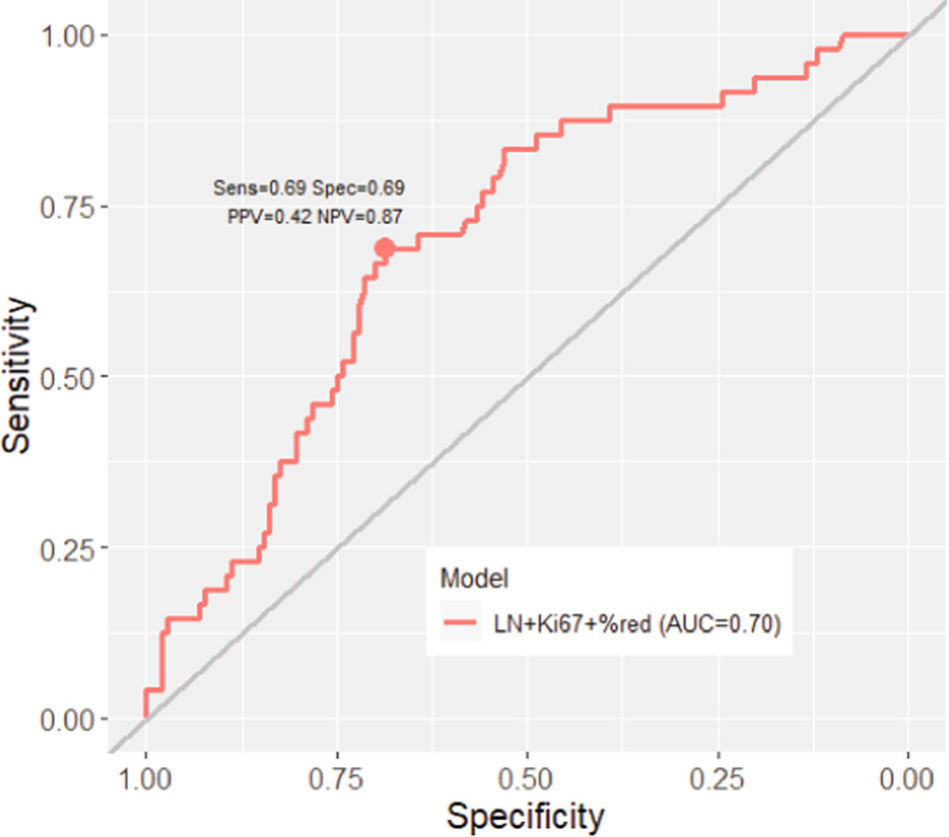
A logistic regression model predictive of PDX engraftment success. The ROC curve is shown for the logistic regression model. The model includes patient’s LN status at diagnosis, % Ki67 positive cells at diagnosis, and mid-NACT tumor volumetric reduction as evaluated by ultrasound.

To generate a model that could predict success in PDX establishment, we selected variables that were modestly univariately associated with PDX success, then created a full logistic regression model followed by stepwise selection to identify the smallest set of explanatory variables. We used Akaike Information Criteria (AIC) for predictor selection. This resulted in a moderately predictive model including three variables (lymph node status at diagnosis, Ki67 score evaluated by IHC of the patient’s diagnostic biopsy, and % tumor volume reduction by ultrasound following AC) with the area under the receiver operating characteristic (ROC) curve equaling 0.7, a positive predictive value of 0.42, and a negative predictive value of 0.87 (Fig. 4). Inclusion of 2-year recurrence status or RCB status at surgery, which were each associated with PDX success in univariate analyses, did not improve the model’s performance. Inclusion of moderately predictive Hallmarks pathway ssGSEA scores did not further improve the model. Thus, a statistical model encompassing diagnostic Ki67 percent positivity, diagnostic lymph node status, and tumor volumetric reduction following front-line AC was able to significantly predict success of PDX engraftment in this large cohort of TNBCs.

## Discussion

Here we report the successful generation of 62 PDX models from the tumors of 217 patients with primary TNBC for an overall success rate of 23%. Patient tumors harbored diverse transcriptomic and histologic features and exhibited a variety of responses to NACT. Overall, the odds of successfully generating a PDX model were significantly higher from TNBCs that were more biologically aggressive and less responsive to NACT: including those that were clinically node positive, Ki67-high, with more extensive residual disease after NACT, and those that recurred within 2 years of surgery. Additional technical variables that may have influenced our success rate in generating PDX models include variable time intervals between tumor cell acquisition and tumor cell engraftment and variation in individual collection techniques resulting in heterogeneous tumor cell yields. In addition, the ability of FNAs to encapsulate the heterogeneity of TNBC is negatively impacted by the spatial heterogeneity associated with TNBC, especially in larger tumors, although we attempted to compensate for this variability by pooling 3 FNAs from different regions of each tumor.

Fifty-one of the 62 PDX models in our collection were derived from the primary breast tumors of patients prior to treatment. Many published PDX collections have been generated from primary or metastatic tumors from previously treated patients. Thus, our collection provides the opportunity to study tumors that have not undergone therapy-induced selection and to identify potential therapies for targeting resistance in the setting of primary, treatment-naïve TNBC. Furthermore, our collection of PDX models were generated from FNAs. FNAs are less painful to patients than are repeated core biopsies and patients are often more likely to consent to serial FNAs. This enabled us to generate serial PDX models from pre- and post-therapy tumors, thereby facilitating future studies aimed at determining how the heterogeneity of TNBC is impacted by NACT as well as identifying targeted therapies to combat chemoresistance in TNBC.

The major goal of this study was to determine which, if any, characteristics of patient tumors predict success in establishing PDX models of TNBC. We did not observe a robust association between gene expression profiles or transcriptomic subtypes with successful PDX engraftment. It is important to note that laboratory-based quality metrics, such as cell number and cell viability from FNAs were not predictive of successful PDX engraftment. The absence of predictive FNA cell metrics motivated our development of a clinical-pathological model to estimate the odds of successful PDX engraftment. Furthermore, PDX engraftment was just as likely to be successful using FNAs from treatment-naive tumors as it was from mid- or post-treatment tumors. So, although the odds of successful engraftment were higher from TNBC tumors that were less sensitive to chemotherapy, the odds of successful engraftment were not higher from mid or post-treatment tumors. This apparent paradox might be related to the loss of cellularity and heterogenous distribution of residual tumor cells within the fibrous tumor bed post-treatment, which impacts the ability to adequately sample residual tumors for PDX generation. In addition, the number of mid-NACT and post-NACT tumors (11) were much less than pre-NACT tumors (51), so estimates of success rates are less precise than those of pre-NACT tumors.

Univariate analyses identified a strong correlation with successful PDX establishment if tumors were used from patients who experienced relapse within 2 years of surgery, and a moderate association was observed with higher RCB status at the time of surgery. A weaker association was observed with low volumetric tumor reduction following AC. In addition, percent Ki67 positivity and lymph node metastasis at the time of diagnosis were both univariately predictive of successful PDX engraftment. Three variables were retained in our multivariable logistic model to predict PDX engraftment. These included diagnostic Ki67 percent positivity, positive diagnostic lymph node status, and low tumor volumetric reduction following front-line AC as determined by ultrasound.

It is well established that RCB status, ascertained by pathologic evaluation of the tumor bed and positive lymph nodes at the time of surgical resection, is associated with metastatic relapse in TNBC. In our cohort, RCB status was only marginally associated with successful PDX engraftment. We observed that early metastatic relapse (within 2 years of surgery) was more strongly predictive of PDX engraftment success, confirming prior findings^22^. Baseline tumor characteristics that were predictive of PDX success included Ki67 positivity and lymph node positivity. Together, these findings suggest that tumor characteristics present at the time of diagnosis, following completion of NACT, and years later upon metastatic relapse, reflect biological features of tumor cells that impact their ability to successfully generate a PDX model.

We analyzed gene expression signatures from 207 treatment-naïve tumor samples to determine if any were predictive of PDX engraftment success. Levels of TILs have been shown to be predictive of NACT response in TNBC patients^28^. PDX engraftment success was not correlated with a TIL gene expression signature or with stromal TIL counts determined by pathology assessment of patients’ diagnostic biopsies. The inherent lack of a fully intact immune system in PDX models could contribute to this lack of correlation. While gene expression profiling of baseline TNBC biopsies was able to only moderately predict PDX success, it is possible that dynamic changes in genomic, transcriptomic, or proteomic features following treatment would be predictive. Indeed, chemotherapy has been demonstrated to impact the genomic and transcriptomic makeup of TNBCs^10,29–31^. Future serial analyses of TNBC biopsies throughout NACT will provide invaluable information to understand the biology of resistance. The models developed herein are a resource for the community to identify biomarkers and to functionally dissect mechanisms of chemoresistance in TNBC.

## Methods

### Patient tissue acquisition

The research conducted in human patients followed all national guidelines including the Health Insurance Portability and Accountability Act privacy and security rules^32^ and the Common Rule (http://www.hhs.gov/ohrp/humansubjects/commonrule/). Patients with newly diagnosed, untreated clinical stage I-III TNBC were eligible for enrollment in ARTEMIS, which was approved and monitored by the Institutional Review Board at The University of Texas, M.D. Anderson Cancer Center (IRB protocol number 2014-0185). All participants provided written informed consent prior to study entry. FNA of the primary breast cancer was obtained under ultrasound guidance prior to treatment (pre-NACT) and three FNA passes were placed into cell culture medium and transported expeditiously to the research laboratory. If there was obvious residual tumor mass following four cycles of Adriamycin combined with cyclophosphamide (mid-NACT) or following all neoadjuvant treatments with taxane ± additional agents (post-NACT), patients could opt for repeat sampling of their residual disease using the same methods. Samples were also collected for histologic or cytologic confirmation and for genomic analyses.

### Patient response evaluation

Patients underwent clinical staging using the AJCC 7th Edition Cancer Staging Manual prior to administration of neoadjuvant therapy. Two-year relapse-free survival was determined based on the standardized definitions for efficacy end points (STEEP) criteria^33^. This was calculated as time in years from surgery until any invasive recurrence of disease (loco-regional or distant). Disease free survival was calculated from the time of surgical resection until the date of initial recurrence, death from any cause, or the date of last documented follow-up if the patient had not developed recurrence or death. Patients without disease recurrence or death were censored from the last date of recorded follow up. Ultrasound response at mid-NACT was assessed by calculating the volumetric reduction of the primary tumor from the pre-NACT volume. Overall response to NACT was determined using the RCB index^1^. Patients who experienced disease progression while receiving NACT and were no longer eligible for curative surgical resection were classified as having RCB-III disease.

### Establishment of orthotopic PDX models

All experimental procedures were approved by the Institutional Animal Care and Use Committee (IACUC) at MD Anderson Cancer Center under IACUC protocol 00000978-RN01. End points for animal experiments were selected in accordance with IACUC approved criteria. 4-week-old female NOD/SCID mice [NOD.CB17-PrkdcScid/NcrCrl, Charles River, National Cancer Institute (NCI) Colony] were used. Mice were housed in modified barrier cages with up to five mice per cage.

PDX models were established according to published protocols^21^. Briefly, the fourth MFPs of 4–5-week-old NOD/SCID female mice were pre-humanized with green fluorescent protein (GFP)–labeled immortalized human mammary stromal fibroblasts (EG cells)^23^ under anesthesia 3–4 weeks prior to tumor cell engraftment. FNA biopsies obtained in the clinic were immediately placed in DMEM/F12 media (HyClone cat# SH30023.01) supplemented with 1 × antibiotic/antimycotic (Corning cat # MT30004CI) and maintained on ice while transported to the lab. Samples were enzymatically digested with complete DMEM/F12 media supplemented with 3 mg/ml collagenase (Roche cat# 1088793) and 250 U/ml of hyaluronidase (Sigma cat# H-3506) and incubated at 37 °C in a rotator for 30–90 min. Following digestion, samples were washed and lysed with red blood cell lysis buffer (Sigma Cat# R7757). Cell pellets (passage 0, P0 cells) were washed and suspended in DMEM/F12 supplemented in 5% bovine calf serum and Matrigel (50/50; Corning cat#354234) before injection into the right fourth MFPs of mice. Under anesthesia, an inverted Y-shaped incision (<0.5 cm) was made adjacent to the nipple of the #4 mammary gland along the thoracic-inguinal region to expose the MFPs of the mice. Tumor cells were injected in a total volume of 30–50 µl. Incisions were closed with wound clips which were removed 7–10 days post-surgery. The typical number of cells implanted in the first generation of mice was 4000–100,000 per MFP depending on the tumor cell yield. In general, two mice per FNA were injected. On average, mice were engrafted with tumor cells 1–2 h after the FNA biopsy was brought to the laboratory.

Engrafted mice were monitored for up to 6 months for tumor growth. Mice exhibiting no palpable tumor within this period were euthanized via CO_2_ inhalation followed by cervical dislocation. When PDX tumors reached ~1000 mm^3^, they were harvested and subjected to four preparations: (1) snap-frozen in liquid nitrogen for quality control tests; (2) fixed in formalin and embedded in paraffin; (3) placed in RNA-Later for sequencing analyses; and (4) dissociated to generate single cell (P1 cells) suspensions. The dissociation of PDX tumors was the same as for FNA biopsy processing, with 3–5 h digestions. P1 tumor cells were immediately implanted into the next generation of mice (*n* = 2), which were not pre-humanized with stromal fibroblasts. Tumors were passaged through a total of three generations of mice (*n* = 6 mice per subsequent generation). When P3 cells were harvested, cryo-preserved cells for re-engraftment, frozen cell pellets, and tumor tissues were banked. PDX models that reached P2 (the 2nd generation of mice) and were validated as described below were considered established. No randomization was necessary as there was only a single group of mice to consider for the establishment of each potential PDX model. The animal research team was blinded to the associated clinical variables until after PDX establishment.

### Validation (Quality control) of PDX models

We used three approaches to validate the identity of each PDX model. First, we confirmed the human origin of each PDX tumor by quantifying the ratio of human-to-mouse genomic DNA in PDX tumors as described previously^12^. Briefly, this was done using qPCR with a human RNaseP gene probe (20× human RNaseP copy number assay, FAM-TAMRA, Life Technologies cat# 4316838) and mouse Trfc gene probe (20× mouse Trfc copy number assay, VIC-TAMRA, Life Technologies). The absolute calibrators were gDNA from TaqMan™ Control Human Genomic DNA (ThermoFisher cat# 4312660) and from Mouse Genomic DNA (Promega cat# G3091). qPCR was conducted using the 2 × TaqMan gene expression master mix (Life Technologies cat# 4369016). The relative ratio of human and mouse gDNA in each tumor sample was calculated using the ΔΔCt method as described previously^12^. PDX tumors consistently exhibiting <10% human genomic DNA were discarded and not included in any subsequent analyses.

Next, to confirm clearance of the GFP-labeled EG cells used to pre-humanize the first generation of mice, we extracted gDNA from PDX tumor cells using the DNeasy Blood & Tissue Kit (Qiagen cat# 69506). Primers amplifying the gene encoding GFP and human GAPDH (positive control), were used in TEMPase 2× hot start polymerase (Apex cat# 5200700-0050) PCR reaction following the protocol of the manufacturer. Amplicons were visualized on agarose gel after electrophoresis. Primer pairs for GFP were: Fwd 5′-AAGTTCATCTGCACCACCG; Rev 5′-TCCTTGAAGAAGATGGTGCG. Primer pairs for GAPDH were: Fwd 5′-ACATCATCCCTGCCTCTAC; Rev 5′-TCAAAGGTGGAGGAGTGG. All PDX models herein were confirmed to be GFP negative by this test.

Lastly, to establish a DNA ‘fingerprint’ for each PDX model for future validation purposes, short-tandem repeat (STR) DNA fingerprinting was conducted using 50 ng of gDNA from P1 and P3 tumor cells through the MDACC Characterized Cell Line Core (CCLC, Cancer Center Support Grant-funded NCI # CA016672). The STR profiles from Promega 16 High Sensitivity STR Kit (cat# DC2100) analyses were compared to commercial databases (DSMZ/ATCC/JCRB/RIKEN) of ~2500 known profiles, as well as the MDACC Characterized Cell Line Core database of ~2000 known DNA fingerprint profiles. Each PDX model had a unique STR profile, indicating none were contaminated with known human cell lines and that no cross-contamination between PDX models had occurred. Matching identities of P1 and P3 PDX samples, as well as PDX samples from serially treated models established from the same patient, showed a consistent profile.

### Histologic analysis of patient biopsies

Core biopsies were obtained from the primary tumor, placed in 10% formalin and paraffin embedded for IHC staining (AR, Ki67, and vimentin). IHC staining for AR, Ki-67 and vimentin was performed on unstained 4 μm-thick tissue sections that had been prepared from a representative paraffin block of tumor in each case using the polymeric biotin-free horseradish peroxidase method on the Leica Microsystems Bond III autostainer (Leica Microsystems, Buffalo Grove, IL, USA). Ki-67 was performed only if the results were not collected from the diagnostic pathology reports. The slides were incubated at 60 °C for 25 min. For AR, following heat-induced epitope retrieval with citrate buffer for 25 min at 100 °C, slides were incubated with mouse monoclonal antibody to AR (clone AR441, Dako 1:30). For Ki-67, following heat-induced epitope retrieval with Tris-EDTA buffer for 20 min at 100 °C, slides were incubated with mouse monoclonal antibody to Ki-67 (clone MIB-1, Dako; 1:100). For vimentin, following heat-induced epitope retrieval with citrate buffer for 5 min at 100 °C, slides were incubated with mouse monoclonal antibody to vimentin (clone V9, 1:900, Dako). The Refine Polymer Detection kit was used to detect bound antibody, with 3,3-diaminobenzidine serving as the chromogen (Leica Microsystems). Slides were counterstained with Mayer’s hematoxylin. Results were evaluated with known positive and negative tissue controls. For AR, the percentage and intensity of any nuclear staining in the tumor cells were recorded. For Ki-67, the percentage of any nuclear staining of any intensity in the tumor cells was recorded. For vimentin, the percentage of moderate or strong cytoplasmic staining in the tumor cells was recorded.

Stromal TILs were assessed on hematoxylin and eosin-stained pre-NACT core biopsy slides based on the International TIL Working Group guidelines whereby percent stromal TILs is calculated as the area of the tumor stroma occupied by mononuclear inflammatory cells divided by the total tumor stromal area^34^. Stromal TILs were evaluated by central pathology review and recorded in increments of 10%, with 5% and above rounded up to the next higher increment.

### Microarray gene expression analysis of patients’ biopsies

Fresh tumor core biopsies (2 cores) were placed into a 1.5 ml vial of RNA*later* (Qiagen) at room temperature, then held in a 4 °C refrigerator (6–72 h) and after that stored frozen at −20 °C until use. Pre-NACT tumor biopsies from the first 200 subjects were delivered to the Molecular Diagnostics Laboratory for prospective testing for the ARTEMIS trial. Pre-NACT tumor biopsies from subsequent subjects in the trial, and all mid-treatment and PDX samples were delivered to a research laboratory and stored at −20 °C until use. RNA was extracted using PicoPure RNA isolation kit (ThermoFisher Scientific, Waltham, MA) following the manufacturer’s instructions. RNA concentration was quantified by Nanodrop (Nanodrop Technologies, Wilmington, DE). The details of the methods for microarray hybridization have been reported previously^35^. Briefly, complementary RNA was prepared from total RNA using the GeneChip 3ʹ IVT PLUS Reagent Kit and hybridized to the U133A Microarray (Thermo Fisher Scientific).

### Subtyping and gene expression analyses

We processed the data using pipelines implemented in the BETSY system^36^. We preprocessed the Affymetrix microarray data using RMA^37^. Using the log_2_ normalized data, we scored an EMT^25^ and plasticity^26^ gene expression signatures using methods previously described^38^. Briefly, we scaled and normalized the expression profile of each gene in the signatures so that they have mean of 0 and variance of 1. Then, we added the normalized values of the genes positively associated with the phenotypes with the additive inverse of the normalized values that were negatively associated. This constituted the signature score for each sample. We also calculated the scores for the Hallmarks pathways^27^ using the implementation of ssGSEA^39^ in the GSVA R library^40^.

Lehmann TNBC subtypes were assigned in the R software environment using the STROMA4 package (Sale and Hallett 2019 STROMA4 R package), after raw intensity files (.CEL) from each microarray were normalized using RMA. For PIK3CA and TILs gene signatures, raw intensity files (.CEL) from each microarray were processed using MAS5.0 to generate probe-level intensities and normalized to a median array intensity of 600, transformed to log2 values, and scaled by the expression levels of 1322 breast cancer reference genes within each sample normalized to median values in a reference cohort^35^. Two PIK3CA-related gene signatures were calculated for each microarray: first, a PIK3CA gene signature that measures transcriptional activity associated with PI3KCA mutation^41^, and secondly, a modification of that first signature that includes only the probe sets robust to technical variation and variation due to tumor heterogeneity^42^. Two TIL gene signatures were calculated for each microarray: a gene signature trained on TIL infiltrate (calculated as the mean gene expression of IGKC, CXCL9, CCL5, and TRBC1 minus the mean gene expression of AK2, APPBP2, ATP5MF, DARS1, LDHA, TRIM2, UBE2Z, UGP2, VDAC2, and WIPF2WIPF2), and a signature to predict high TILs after subsequent neoadjuvant chemotherapy^43^.

### Statistical analyses

Interval scale baseline patient characteristics, tumor characteristics and molecular signature scores were summarized descriptively with means and standard deviations and compared univariately by PDX success status using *t*-tests. Categorical patient characteristics, tumor characteristics and molecular categories were summarized with counts and proportions and compared univariately by PDX success status using Fisher’s exact test. For pre-NACT biopsies, multiple logistic regression was used to combine features that were at least modestly univariately associated with PDX success into a single model. We used stepwise (forward and backward) selection based on the Akaike Information Criterion (AIC) to identify the smallest useful set of explanatory variables. Area under the corresponding ROC curve was used to summarize the final model. Diagnostic characteristics such as positive predictive value and negative predictive value were estimated at the Youden index cut-point. Analyses were conducted using R (v3.6.0-4.1.2) and packages tableone^44^, vtree, pROC, pheatmap, ggplot2^45^, and cutpointr.

### Reporting summary

Further information on research design is available in the Nature Research Reporting Summary linked to this article.

## Supplementary information

Supplementary Information

Supplementary Table 2

Reporting Summary

## Data Availability

Deidentified gene expression data generated in this study is deposited in the Gene Expression Omnibus (GEO) database under accession number GSE216333. PDX tumors are available upon request to H.P.-W. under a material transfer agreement with the University of Texas MDACC.

## Code availability

All codes used in this study are standard as detailed in the methods section and will be shared upon request.
