# Supplementary material for: Predictors of success in establishing orthotopic patient-derived xenograft models of triple negative breast cancer
Source: NPJ Breast Cancer. 2023 Jan 10;9:2. doi: 10.1038/s41523-022-00502-1 (PMC9831981; doi:10.1038/s41523-022-00502-1)
Supplement: Supplementary file 1 — Supplementary Information [file 41523_2022_502_MOESM1_ESM.pdf]

Supplementary Figure 1

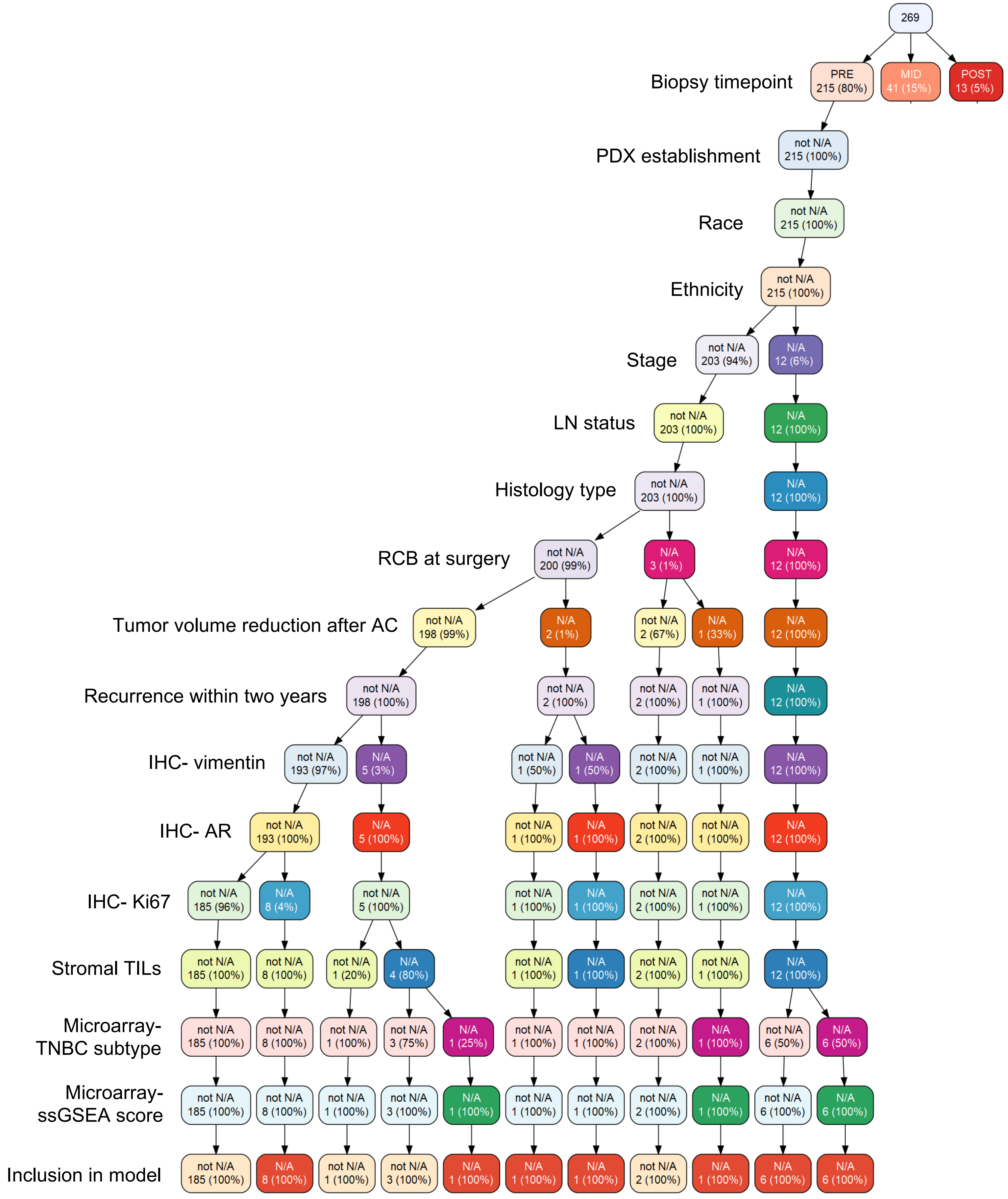

Supplementary Figure 1. Consort diagram. The number of biopsies evaluated for each variable are indicated in each box. N/a indicates data was not available for a given variable.

Supplementary Table 1

PDX model name	Patient biopsy treatment stage	patient FNA number of viable cells	Successful PDX? (0=no, 1=yes)	STR fingerprinting profile	GFP PCR	%human vs mouse qPCR average
PIM009	PRE	1.85E+04	0			
PIM010	PRE	1.50E+06	1	Unique	Negative	58.0%
PIM011	PRE	1.40E+05	0			
PIM012	PRE	1.78E+06	0			
PIM013	PRE	1.00E+05	1	Unique	Negative	50.8%
PIM014	PRE	1.45E+05	1	Matched PIM024, PIM051	Negative	68.7%
PIM016	PRE	4.50E+04	0			
PIM017	MID	1.89E+05	0			
PIM018	PRE	8.00E+04	0			
PIM019	PRE	3.10E+04	0			
PIM022	PRE	1.75E+04	0			
PIM023	PRE	2.06E+05	0			
PIM024	MID	4.50E+05	1	Matched PIM014, PIM051	Negative	70.0%
PIM025	PRE	1.06E+05	1	Matched PIM038	Negative	69.7%
PIM027	PRE	4.00E+05	0			
PIM028	PRE	4.25E+04	1	Unique	Negative	67.7%
PIM029	PRE	1.90E+05	0			
PIM030	PRE	1.10E+05	0			
PIM031	PRE	1.10E+04	0			
PIM032	PRE	1.10E+04	0			
PIM033	PRE	6.50E+04	0			
PIM034	PRE	7.00E+04	0			
PIM035	MID	4.50E+04	0			
PIM036	PRE	3.90E+05	0			
PIM037	PRE	4.67E+04	0			
PIM038	MID	4.82E+05	1	Matched PIM025	Negative	64.0%
PIM039	PRE	2.13E+04	0			
PIM040	PRE	1.33E+05	1	Unique	Negative	80.7%
PIM041	PRE	7.25E+05	0			
PIM042	PRE	2.60E+05	1	Unique	Negative	78.7%
PIM043	PRE	1.50E+05	0			
PIM044	PRE	2.90E+05	0			
PIM045	PRE	5.40E+04	0			
PIM046	PRE	7.60E+04	1	Unique	Negative	73.1%
PIM047	PRE	1.30E+05	0			
PIM048	PRE	3.60E+04	1	Unique	Negative	73.8%

PIM049	PRE	1.20E+06	1	Unique	Negative	45.3%
PIM050	MID	4.90E+04	1	Unique	Negative	30.0%
PIM051	POST	4.00E+04	1	Matched PIM014, PIM024	Negative	81.9%
PIM052	PRE	2.60E+05	0			
PIM053	PRE	5.50E+05	1	Unique	Negative	82.8%
PIM054	PRE	2.00E+05	0			
PIM055	PRE	1.40E+05	0			
PIM056	PRE	2.00E+05	1	Unique	Negative	32.5%
PIM057	PRE	1.30E+05	0			
PIM058	POST	1.30E+04	0			
PIM059	PRE	1.40E+05	0			
PIM060	PRE	1.10E+06	0			
PIM061	MID	5.40E+04	0			
PIM062	PRE	1.00E+06	0			
PIM063	PRE	5.90E+05	0			
PIM064	PRE	6.80E+05	0			
PIM065	MID	1.28E+04	0			
PIM066	PRE	1.96E+05	1	Unique	Negative	48.4%
PIM067	PRE	4.80E+04	0			
PIM068	PRE	1.28E+04	1	Unique	Negative	72.9%
PIM069	PRE	4.80E+04	0			
PIM070	MID	4.28E+04	0			
PIM071	PRE	2.50E+05	0			
PIM072	PRE	1.13E+04	0			
PIM073	PRE	1.18E+06	1	Unique	Negative	76.6%
PIM074	PRE	2.11E+05	0			
PIM075	PRE	1.40E+05	0			
PIM076	PRE	1.40E+05	0			
PIM077	PRE	1.10E+05	1	Matched PIM110	Negative	69.8%
PIM078	PRE	2.40E+05	0			
PIM079	PRE	1.35E+05	0			
PIM080	PRE	1.50E+05	0			
PIM081	PRE	3.70E+05	0			
PIM082	PRE	4.68E+04	0			
PIM083	PRE	6.45E+04	0			
PIM084	PRE	1.94E+05	1	Unique	Negative	52.2%
PIM085	PRE	1.93E+05	0			
PIM086	PRE	4.40E+04	0			
PIM087	PRE	5.75E+05	0			
PIM088	PRE	1.39E+05	0			
PIM089	PRE	1.53E+05	0			
PIM090	PRE	1.28E+04	0			
PIM091	PRE	4.60E+04	1	Unique	Negative	77.3%

PIM092	PRE	4.45E+04	0			
PIM093	PRE	1.16E+04	0			
PIM094	PRE	7.40E+03	0			
PIM095	PRE	2.75E+05	0			
PIM096	MID	1.79E+05	0			
PIM097	PRE	6.80E+05	1	Unique	Negative	44.7%
PIM098	PRE	1.85E+04	0			
PIM099	PRE	1.49E+05	1	Unique	Negative	48.3%
PIM100	PRE	1.71E+04	0			
PIM101	PRE	1.74E+05	0			
PIM102	PRE	1.56E+04	0			
PIM103	PRE	4.32E+04	0			
PIM104	PRE	8.50E+05	0			
PIM105	PRE	1.00E+06	0			
PIM106	PRE	8.40E+05	1	Unique	Negative	59.4%
PIM107	PRE	6.75E+05	0			
PIM108	MID	3.13E+04	0			
PIM110	MID	7.88E+03	1	Matched PIM077	Negative	73.5%
PIM111	MID	7.00E+03	0			
PIM112	PRE	1.90E+05	0			
PIM113	PRE	2.10E+05	0			
PIM114	POST	7.40E+03	1	Unique	Negative	63.2%
PIM115	PRE	6.10E+05	0			
PIM116	PRE	8.50E+03	0			
PIM117	PRE	1.57E+05	1			
PIM118	PRE	3.38E+04	0			
PIM119	PRE	4.75E+04	0			
PIM120	PRE	2.28E+04	0			
PIM121	MID	4.00E+04	0			
PIM122	MID	2.72E+04	0			
PIM123	PRE	1.22E+05	0			
PIM124	PRE	1.93E+04	0			
PIM125	MID	8.50E+03	0			
PIM126	PRE	1.62E+05	0			
PIM127	PRE	1.45E+05	0			
PIM128	MID	2.50E+04	0			
PIM129	PRE	4.50E+04	0			
PIM130	MID	4.00E+04	0			
PIM131	PRE	1.43E+04	0			
PIM132	PRE	2.81E+05	0			
PIM133	PRE	7.30E+04	0			
PIM134	PRE	3.66E+04	0			
PIM135	PRE	1.99E+04	1	Unique	Negative	79.8%

PIM136	PRE	7.25E+02	0			
PIM137	PRE	1.70E+04	1	Matched PIM172	Negative	66.3%
PIM138	PRE	1.06E+04	0			
PIM140	POST	4.20E+05	0			
PIM141	PRE	3.50E+04	0			
PIM142	PRE	8.57E+04	0			
PIM143	PRE	3.50E+04	0			
PIM144	PRE	8.20E+04	0			
PIM145	MID	1.34E+04	0			
PIM146	PRE	5.65E+04	0			
PIM147	PRE	2.77E+04	1	Unique	Negative	80.8%
PIM148	PRE	2.02E+05	0			
PIM149	PRE	9.00E+04	0			
PIM150	PRE	2.40E+04	0			
PIM151	PRE	3.95E+04	0			
PIM152	PRE	4.28E+03	0			
PIM153	PRE	1.90E+04	0			
PIM154	PRE	3.80E+04	0			
PIM156	PRE	7.60E+04	0			
PIM157	PRE	3.70E+04	0			
PIM158	MID	7.90E+03	0			
PIM159	MID	3.23E+04	0			
PIM160	PRE	4.10E+04	0			
PIM161	PRE	2.00E+04	1	Unique	Negative	44.7%
PIM162	PRE	9.70E+04	0			
PIM163	PRE	4.70E+04	1	Unique	Negative	82.8%
PIM164	PRE	2.15E+05	1	Unique	Negative	90.3%
PIM165	PRE	1.25E+04	1	Unique	Negative	61.6%
PIM166	PRE	7.00E+03	1	Unique	Negative	91.9%
PIM167	PRE	1.34E+04	0			
PIM168	PRE	7.80E+03	0			
PIM169	PRE	3.67E+04	0			
PIM170	POST	2.27E+04	0			
PIM171	PRE	2.80E+04	1	Unique	Negative	60.4%
PIM172	MID	1.29E+04	1	Matched PIM137	Negative	53.1%
PIM173	PRE	8.00E+04	1	Unique	Negative	78.2%
PIM174	MID	1.27E+04	0			
PIM175	MID	8.55E+03	0			
PIM177	PRE	1.38E+05	0			
PIM178	PRE	2.13E+04	0			
PIM179	PRE	2.06E+04	0			
PIM181	PRE	1.00E+06	1	Unique	Negative	33.2%
PIM182	PRE	1.29E+05	1	Unique	Negative	52.9%

PIM183	MID	7.80E+03	0			
PIM184	PRE	1.70E+04	0			
PIM185	PRE	3.30E+04	0			
PIM186	POST	1.13E+04	0			
PIM187	POST	1.98E+04	0			
PIM188	MID	2.25E+05	0			
PIM189	MID	4.72E+04	0			
PIM190	PRE	1.57E+05	1	Matched PIM231	Negative	44.9%
PIM191	PRE	9.30E+03	1	Unique	Negative	40.9%
PIM193	PRE	2.65E+04	0			
PIM194	PRE	1.86E+04	0			
PIM195	PRE	7.10E+03	1	Unique	Negative	61.1%
PIM196	MID	5.70E+03	0			
PIM197	PRE	1.35E+04	0			
PIM198	PRE	3.00E+05	0			
PIM199	MID	9.00E+04	0			
PIM200	PRE	8.74E+04	0			
PIM202	PRE	2.60E+04	0			
PIM203	PRE	5.70E+03	0			
PIM204	MID	1.40E+03	0			
PIM205	POST	3.20E+04	0			
PIM206	PRE	9.30E+03	0			
PIM207	PRE	2.13E+04	0			
PIM208	PRE	2.57E+04	0			
PIM209	PRE	2.26E+04	0			
PIM210	MID	2.58E+04	0			
PIM211	PRE	3.40E+04	0			
PIM212	PRE	1.00E+06	0			
PIM213	PRE	2.94E+05	0			
PIM214	PRE	5.00E+04	1	Unique	Negative	72.7%
PIM215	PRE	3.60E+05	1	Unique	Negative	56.5%
PIM216	MID	2.56E+04	0			
PIM217	POST	1.99E+04	0			
PIM218	PRE	8.34E+04	0			
PIM219	PRE	5.86E+04	0			
PIM220	PRE	1.85E+04	0			
PIM221	MID	1.13E+04	0			
PIM222	PRE	4.00E+04	0			
PIM223	POST	3.60E+04	0			
PIM224	PRE	2.49E+04	1	Unique	Negative	87.3%
PIM225	PRE	7.90E+04	1	Unique	Negative	36.1%
PIM227	PRE	5.36E+05	0			
PIM228	PRE	3.52E+04	1	Unique	Negative	25.7%

PIM229	POST	3.30E+03	0			
PIM230	PRE	1.10E+04	0			
PIM231	MID	3.50E+04	1	Matched PIM190	Negative	38.5%
PIM232	PRE	4.40E+04	0			
PIM234	PRE	6.60E+04	0			
PIM235	PRE	3.55E+05	0			
PIM236	MID	7.15E+04	0			
PIM237	PRE	2.70E+04	0			
PIM238	PRE	2.49E+04	0			
PIM239	PRE	3.50E+05	1	Unique	Negative	36.8%
PIM240	PRE	1.65E+04	0			
PIM241	PRE	6.50E+03	0			
PIM242	PRE	8.63E+03	1	Matched PIM269	Negative	66.2%
PIM243	MID	2.85E+03	0			
PIM245	PRE	3.46E+04	1	Unique	Negative	82.2%
PIM246	PRE	1.12E+05	0			
PIM248	PRE	2.20E+04	0			
PIM249	PRE	8.90E+03	0			
PIM250	PRE	3.95E+04	0			
PIM251	MID	4.35E+04	0			
PIM252	PRE	6.26E+04	0			
PIM253	PRE	1.11E+05	0			
PIM254	PRE	1.92E+04	1	Matched PIM311	Negative	58.7%
PIM255	MID	1.56E+04	0			
PIM256	PRE	1.20E+05	0			
PIM259	MID	1.66E+04	0			
PIM260	PRE	2.84E+04	0			
PIM261	PRE	1.34E+04	0			
PIM262	PRE	1.54E+05	1	Matched PIM284, PIM321	Negative	71.5%
PIM265	PRE	2.15E+05	0			
PIM267	PRE	8.60E+04	0			
PIM268	PRE	2.34E+04	0			
PIM269	MID	1.86E+04	1	Matched PIM242	Negative	72.2%
PIM271	PRE	1.67E+04	0			
PIM272	PRE	1.70E+04	0			
PIM273	PRE	3.00E+04	0			
PIM274	MID	1.42E+04	0			
PIM275	PRE	8.00E+04	0			
PIM276	PRE	2.12E+04	0			
PIM277	PRE	4.96E+04	0			
PIM278	PRE	2.80E+05	0			
PIM279	PRE	9.60E+04	0			
PIM280	PRE	3.90E+04	0			

PIM281	PRE	1.80E+04	0			
PIM282	PRE	4.30E+04	0			
PIM283	PRE	1.45E+05	1	Unique	Negative	70.0%
PIM284	MID	4.30E+04	1	Matched PIM262, PIM321	Negative	79.1%
PIM285	PRE	2.80E+04	1	Unique	Negative	36.8%
PIM287	PRE	4.00E+04	0			
PIM289	PRE	3.20E+04	0			
PIM290	PRE	4.18E+04	0			
PIM291	POST	4.10E+04	1	Unique	Negative	81.5%
PIM293	PRE	1.45E+04	0			
PIM294	POST	9.25E+03	0			
PIM295	PRE	1.00E+05	0			
PIM296	PRE	5.80E+04	0			
PIM297	PRE	2.16E+05	0			
PIM298	PRE	4.10E+05	0			
PIM300	PRE	1.55E+05	1	Unique	Negative	86.8%
PIM301	PRE	3.20E+03	0			
PIM302	PRE	6.30E+04	1	Unique	Negative	64.4%
