# Supplementary material for: Predictors of success in establishing orthotopic patient-derived xenograft models of triple negative breast cancer
Source: NPJ Breast Cancer. 2023 Jan 10;9:2. doi: 10.1038/s41523-022-00502-1 (PMC9831981; doi:10.1038/s41523-022-00502-1)
Supplement: Supplementary file 3 — Reporting Summary [file 41523_2022_502_MOESM3_ESM.pdf]

nature portfolio

Corresponding author(s): Helen Piwnica-Worms, Gloria Echeverria

Last updated by author(s): Aug 9, 2022

Reporting Summary

Nature Portfolio wishes to improve the reproducibility of the work that we publish. This form provides structure for consistency and transparency in reporting. For further information on Nature Portfolio policies, see our Editorial Policies and the Editorial Policy Checklist.

Statistics

For all statistical analyses, confirm that the following items are present in the figure legend, table legend, main text, or Methods section.

n/a	Confirmed	
☐	☒	The exact sample size (*n*) for each experimental group/condition, given as a discrete number and unit of measurement
☐	☒	A statement on whether measurements were taken from distinct samples or whether the same sample was measured repeatedly
☐	☒	The statistical test(s) used AND whether they are one- or two-sided<br>*Only common tests should be described solely by name; describe more complex techniques in the Methods section.*
☐	☒	A description of all covariates tested
☐	☒	A description of any assumptions or corrections, such as tests of normality and adjustment for multiple comparisons
☐	☒	A full description of the statistical parameters including central tendency (e.g. means) or other basic estimates (e.g. regression coefficient) AND variation (e.g. standard deviation) or associated estimates of uncertainty (e.g. confidence intervals)
☐	☒	For null hypothesis testing, the test statistic (e.g. *F*, *t*, *r*) with confidence intervals, effect sizes, degrees of freedom and *P* value noted<br>*Give P values as exact values whenever suitable.*
☐	☒	For Bayesian analysis, information on the choice of priors and Markov chain Monte Carlo settings
☐	☒	For hierarchical and complex designs, identification of the appropriate level for tests and full reporting of outcomes
☐	☒	Estimates of effect sizes (e.g. Cohen's *d*, Pearson's *r*), indicating how they were calculated

Our web collection on statistics for biologists contains articles on many of the points above.

Software and code

Policy information about availability of computer code

Data collection	Analyses were conducted using R (v3.6.0-4.1.2) and packages tableone39, vtree, pROC, pheatmap, ggplot240, and cutpointr.
Data analysis	Analyses were conducted using R (v3.6.0-4.1.2) and packages tableone39, vtree, pROC, pheatmap, ggplot240, and cutpointr. Lehmann TNBC subtypes were assigned in the R software environment using the STROMA4 package (Sale and Hallett 2019 STROMA4 R package), after raw intensity files (.CEL) from each microarray were normalized using RMA. For PIK3CA and TILs gene signatures, raw intensity files (.CEL) from each microarray were processed using MAS5.0 to generate probe-level intensities and normalized to a median array intensity of 600, transformed to log2 values, and scaled by the expression levels of 1322 breast cancer reference genes within each sample normalized to median values in a reference cohort27. Two PIK3CA-related gene signatures were calculated for each microarray: first, a PIK3CA gene signature that measures transcriptional activity associated with PI3KCA mutation36, and secondly, a modification of that first signature that includes only the probe sets robust to technical variation and variation due to tumor heterogeneity37. Two TIL gene signatures were calculated for each microarray: a 6-gene signature trained on TIL infiltrate (Sinn BV et al 2022 manuscript in preparation), and a signature to predict high TILs after subsequent neoadjuvant chemotherapy38.

For manuscripts utilizing custom algorithms or software that are central to the research but not yet described in published literature, software must be made available to editors and reviewers. We strongly encourage code deposition in a community repository (e.g. GitHub). See the Nature Portfolio guidelines for submitting code & software for further information.

Data

Policy information about availability of data

All manuscripts must include a data availability statement. This statement should provide the following information, where applicable:

- Accession codes, unique identifiers, or web links for publicly available datasets
- A description of any restrictions on data availability
- For clinical datasets or third party data, please ensure that the statement adheres to our policy

Deidentified gene expression data generated in this study will be deposited in the Gene Expression Omnibus (GEO) database upon acceptance of this manuscript for publication. PDX tumors are available upon request to H.P.-W. under a material transfer agreement with the University of Texas MDACC.

Human research participants

Policy information about studies involving human research participants and Sex and Gender in Research.

Reporting on sex and gender	All patients enrolled in this study were female as this was a breast cancer clinical trial
Population characteristics	Relevant population characteristics are listed in Table 1.
Recruitment	Patients with newly diagnosed, untreated clinical stage I-III TNBC were eligible for enrollment in ARTEMIS, which was approved and monitored by the Institutional Review Board at The University of Texas, M.D. Anderson Cancer Center (IRB protocol number 2014-0185).
Ethics oversight	The research conducted in human patients followed all national guidelines including the Health Insurance Portability and Accountability Act (HIPAA) privacy and security rules23 and the Common Rule (http://www.hhs.gov/ohrp/humansubjects/commonrule/). Patients with newly diagnosed, untreated clinical stage I-III TNBC were eligible for enrollment in ARTEMIS, which was approved and monitored by the Institutional Review Board at The University of Texas, M.D. Anderson Cancer Center (IRB protocol number 2014-0185). All participants provided written informed consent prior to study entry.

Note that full information on the approval of the study protocol must also be provided in the manuscript.

Field-specific reporting

Please select the one below that is the best fit for your research. If you are not sure, read the appropriate sections before making your selection.

☒ Life sciences ☐ Behavioural & social sciences ☐ Ecological, evolutionary & environmental sciences

For a reference copy of the document with all sections, see nature.com/documents/nr-reporting-summary-flat.pdf

Life sciences study design

All studies must disclose on these points even when the disclosure is negative.

Sample size	A sample size of 217 patients was included in this study. These were the first 217 patients enrolled in the ARTEMIS clinical trial from whom samples were obtained for PDX engraftment.
Data exclusions	Disease free survival was calculated from the time of surgical resection until the date of initial recurrence, death from any cause, or the date of last documented follow-up if the patient had not developed recurrence or death. Patients without disease recurrence or death were censored from the last date of recorded follow up. Of the 217 ARTEMIS patients biopsied for PDX establishment, 207 pre-NACT biopsies passed quality control for Affymetrix gene expression microarray analysis.
Replication	na
Randomization	na
Blinding	All investigators collecting patient data and running statistical analyses against PDX data were blinded to sample identity.

Reporting for specific materials, systems and methods

We require information from authors about some types of materials, experimental systems and methods used in many studies. Here, indicate whether each material, system or method listed is relevant to your study. If you are not sure if a list item applies to your research, read the appropriate section before selecting a response.

Materials & experimental systems

n/a	Involved in the study
☐	☒ Antibodies
☒	☐ Eukaryotic cell lines
☒	☐ Palaeontology and archaeology
☐	☒ Animals and other organisms
☐	☒ Clinical data
☒	☐ Dual use research of concern

Methods

n/a	Involved in the study
☒	☐ ChIP-seq
☒	☐ Flow cytometry
☒	☐ MRI-based neuroimaging

Antibodies

Antibodies used	Androgen Receptor (clone AR441, Dako, Carpinteria, CA, USA; 1:30); Ki-67 (clone MIB-1, Dako; 1:100); vimentin (clone V9, 1:900, Dako).
Validation	all have been validated on human patient tumor specimens

Animals and other research organisms

Policy information about studies involving animals; ARRIVE guidelines recommended for reporting animal research, and Sex and Gender in Research

Laboratory animals	Female NOD/SCID mice [NOD.CB17-PrkdcScid/NcrCrl, Charles River, National Cancer Institute (NCI) Colony] were obtained from Charles River.
Wild animals	na
Reporting on sex	All mice used in this study were female, as all human breast cancer patients whose samples were used to generate PDX in this study from the clinical trial were also female.
Field-collected samples	na
Ethics oversight	All experimental procedures were approved by the Institutional Animal Care and Use Committee (IACUC) at MD Anderson Cancer Center under IACUC protocol 00000978-RN01. End points for animal experiments were selected in accordance with IACUC approved criteria.

Note that full information on the approval of the study protocol must also be provided in the manuscript.

Clinical data

Policy information about clinical studies

All manuscripts should comply with the ICMJE guidelines for publication of clinical research and a completed CONSORT checklist must be included with all submissions.

Clinical trial registration	NCT02276443
Study protocol	NCT02276443 clinicaltrials.gov
Data collection	We generated PDX models from the tumors of TNBC patients enrolled on a neoadjuvant clinical trial, ARTEMIS (A Robust TNBC Evaluation fraMework to Improve Survival (NCT02276443)). Biopsies were obtained by fine-needle aspiration (FNA) prior to treatment with chemotherapy ('pre'), following four cycles of Adriamycin combined with cyclophosphamide (AC; 'mid'), and following treatment with a taxane, sometimes combined with an experimental targeted therapy prior to surgery ('post'). We catalogued clinical information associated with each patient biopsy and conducted statistical analyses to search for parameters associated with successful PDX engraftment and we demonstrated that FNAs can be used to generate PDX models.
Outcomes	The research conducted in human patients followed all national guidelines including the Health Insurance Portability and Accountability Act (HIPAA) privacy and security rules23 and the Common Rule (http://www.hhs.gov/ohrp/humansubjects/commonrule/). Patients with newly diagnosed, untreated clinical stage I-III TNBC were eligible for enrollment in ARTEMIS, which was approved and monitored by the Institutional Review Board at The University of Texas, M.D. Anderson Cancer Center (IRB protocol number 2014-0185). All participants provided written informed consent prior to study entry. Patients underwent clinical staging using the AJCC 7th Edition Cancer Staging Manual prior to administration of neoadjuvant therapy. Two-year relapse-free survival was determined based on the standardized definitions for efficacy end points (STEEP) criteria24. This was calculated as time in years from surgery until any invasive recurrence of disease (loco-regional or distant). Disease free survival was calculated from the time of surgical resection until the date of initial recurrence, death from any cause, or the date of last documented follow-up if the patient had not developed recurrence or death. Patients without disease recurrence or death were censored from the last date of recorded follow up. Ultrasound response at mid-NACT was assessed by calculating the volumetric reduction of the primary tumor from the pre-NACT volume. Overall response to NACT was determined using the RCB index1. Patients who experienced disease progression while receiving NACT and were no longer eligible for curative surgical resection were classified as having RCB-III disease.
